# A Critical Mutualism – Competition Interplay Underlies the Loss of Microbial Diversity in Sedentary Lifestyle

**DOI:** 10.3389/fmicb.2019.03142

**Published:** 2020-01-22

**Authors:** Nazareth Castellanos, Gustavo G. Diez, Carmen Antúnez-Almagro, María Bailén, Carlo Bressa, Rocío González Soltero, Margarita Pérez, Mar Larrosa

**Affiliations:** ^1^Nirakara Lab, Institute of Research and Cognitive Science, Madrid, Spain; ^2^Dementia Care Unit, University Hospital Virgen de la Arrixaca, Murcia, Spain; ^3^Masmicrobiota Group, Faculty of Biomedical and Health Sciences, Universidad Europea de Madrid, Madrid, Spain; ^4^Faculty of Sport and Health Sciences, Universidad Europea de Madrid, Madrid, Spain

**Keywords:** gut microbiota, active lifestyle, microbial interactions, microbiota network, sedentarism

## Abstract

Physical exercise improves the overall health status by preventing the development of several diseases. In recent years, it has been observed that physical exercise impacts gut microbiota by increasing the presence of beneficial bacteria and microbial diversity. In contrast, a sedentary lifestyle increases the incidence of chronic diseases that often have an associated loss of microbial diversity. The gut microbiota is a vast ecosystem in which microorganisms interact with each other in different ways; however, microbial ecosystem interactions are scarcely studied. The goal of this study was to determine whether individuals with a sedentary lifestyle have lower diversity in their gut microbiota and how microbial diversity is associated with changes in bacterial network interactions. For that purpose, diet, body composition, physical activity, and sedentarism behavior were characterized for individuals who did or did not comply with the World Health Organization recommendations for physical activity. The composition of the gut microbiome was determined by 16S rRNA gene sequencing. Reorganization of microbial structure with lifestyle was approached from network analysis, where network complexity and the topology of positive and negative interdependences between bacteria were compared and correlated with microbial diversity. Sedentary lifestyle was significantly associated with a diet low in fiber and rich in sugars and processed meat, as well as with high visceral and total corporal fat composition. The diversity (phylogenic diversity, Chao, observed species, and Shannon’s index) and network complexity of the gut microbiota were significantly lower in sedentary compared to active individuals. Whereas mutualism or co-occurrence interactions were similar between groups, competitiveness was significantly higher in the active lifestyle group. The mutualism-competition ratio was moderate and positively associated with diversity in sedentary individuals, but not in active individuals. This finding indicates that there is a critical point in this ratio beyond which the stability of the microbial community is lost, inducing a loss of diversity.

## Introduction

The gut microbiota has emerged as an important factor in human health and disease. Indeed, specific microbial patterns have been associated with numerous disorders, such as cancer or cardiovascular disease (Marchesi et al., 2011; Jie et al., 2017). Numerous studies that compare the gut microbiota in health and disease point to the loss of microbial diversity as a disease characteristic. For instance, patients with ileal Crohn’s disease, irritable bowel syndrome or rheumatoid arthritis, have lower microbiota diversity compared to their healthy peers (Jeffery et al., 2011; Imhann et al., 2016). Microbiota diversity has also been associated with disease severity (McMurtry et al., 2015), and correlates with immune system parameters (Ribeiro et al., 2017). The physiological state of the host is also associated with microbiota composition, and aging and obesity are connected with decreased microbiota richness (Ley et al., 2006b; Turnbaugh et al., 2008; Claesson et al., 2012). These associations may be due to lifestyle characteristics that govern the gut microbial community, diets rich in sugars and/or fats, or the loss of mastication efficiency that leads to a modification of the diet or antibiotic intake, among others. However, the underlying mechanisms through which this loss of diversity occurs and the effects on the structure of the microbial community remain unclear. From an ecological perspective, microbial communities with greater diversity are more stable, resistant to pathogenic invasions, exhibit a higher capacity for recovery, and show functional redundancy that leads to more efficient utilization of resources. Together, these effects promote health benefits for the host (Lozupone et al., 2012; Karkman et al., 2017).

The gut microbiota is a complex ecosystem characterized by its composition, as well as the interactions among species that shape the microbial community. Most previous studies focused on the estimation of cooperation strength among species, under the assumption that mutualism is the driving force of diversity structure (Rejmánek and Starý, 1979). Consequently, they underevaluate competition (Gracia-Lázaro et al., 2018). It is now well accepted, however, that positive and negative interactions define the community and ecosystem (Agrawal, 2007; Mougi and Kondoh, 2012; Suweis et al., 2013). Thus, diversity is based on both competition and mutualism networks. Notwithstanding the great advancements in sequencing technologies s made to reveal the structure and variations of the microbiota network and its relationship with the host, this task remains challenging.

To understand the relationship between the microbiota and the host, it is necessary to know both the composition of the gut microbiota and the interactions that define the microbiota network. Additionally, structure and function of the bacterial communities are critically important. Understanding the interactions will help develop treatments or modifications to the microbiota with the aim of exerting a favorable effect on the host. Indeed, several strategies have been utilized to improve host health via microbiota modification, including the use of probiotics, prebiotics, and fecal transplants, but how these treatments impact the microbial community is not known with any certainty. For these reasons, it is crucial to define interactions [cooperation or mutualism (co-occurrence) and competition (co-exclusion)] between microbial community members to understand the community response to external factors (diet, antibiotics and physical activity, among others (Shade et al., 2012). The presence, abundance, and metabolism of bacteria strongly depends on the complex interactions between them; these interactions are positive in cases of co-occurrence (cooperation or mutualism), negative in case of competition, or null in cases of no impact. Mutualism (positive relationship between bacterial abundances) can be due to cross-feeding, co-aggregation in biofilms, co-colonization, niche overlap or other reasons. Competition (negative relationship between bacterial abundances) may result from amensalism or a prey– predator relationship. The study of microbial interactions provides additional information to species abundance; network analysis represents a promising perspective to study microbial community stability (see review Faust and Raes, 2012). Of special interest is the relationship between mutualism and competition networks in different conditions; this factor determines species coexistence.

The prevalence of physical inactivity has increased alarmingly, and sedentary lifestyle has emerged as a risk factor for obesity and several other disorders, such as cancer and cardiovascular disease (Packey and Ciorba, 2010; Winzer et al., 2011; Chomistek et al., 2013; Gonzalez-Gross and Melendez, 2013). Sedentary lifestyle and lack of physical activity are also associated with a less healthy dietary pattern (Loprinzi, 2016), and combination of these factors potentiates the negative effect on health (Laxer et al., 2017). Given these considerations, one can assume that individuals who exhibit sedentary behavior, a low level of physical activity, and an unhealthy diet, are more prone to disease.

The aim of this work was to study the mechanisms that underlie the influence of lifestyle on microbial diversity from a new network analysis perspective, with a focus on the interactions among microbial taxa.

## Materials and Methods

### Participants Characteristics

This observational, transversal study involved free-living individuals. Volunteers were recruited using posters, social networks, and magazines. The study was performed at Universidad Europea de Madrid (Madrid, Spain). A total of 143 otherwise healthy non-smoking individuals were enrolled in the study according to the following inclusion criteria: age 18–40 years and body mass index (BMI) of 20–30 kg/m^2^. Exclusion criteria were any change in diet during the previous year, any kind of pathology, previous gastrointestinal surgery, antibiotic intake during the 3 months prior to the study, smoking, prebiotics, probiotics, vegetarian or vegan dietary pattern, nutritional or ergogenic complements (caffeine, conjugated linoleic acid, hydroxymethylbutyrate, carnitine, creatine, proteins, ginseng, glutamine, and ribose et cetera), pregnancy, or lactation. In order to divide the sample into active and sedentary individuals, volunteers were asked about their exercise habits during the preceding year, prior to the start of the study. Criteria for physical activity were as follows: sedentary individuals were those who did not perform the minimal physical activity established as healthy by the World health organization (WHO), that is, 3 days of exercise per week for 30 min at a moderate intensity (bicycling at a regular pace, swimming or other fitness activities; World Health Organization, 2010). Active individuals were those who performed at least 3 h of physical exercise per week. According to these criteria from the 143 volunteers, 109 were selected for the study, and 34 were excluded because they did not have a clearly active or sedentary lifestyle but alternated periods of physical activity with episodes when they exercised less, or fulfilled one of the exclusion criteria. Of the 109 individuals, 64 individuals were classified as active (ACT; 37 males/27 females) and 45 (28 males/17 females) as sedentary (SED). All individuals were Caucasian. The Ethics Committee for Clinical Research of the Ramón y Cajal Hospital CEIC 338-14 (Madrid, Spain) approved the study. All procedures were in accordance with the 1964 Declaration of Helsinki and its later amendments. All participants received written full disclosure of the relevant project information, including the purpose of the trial, the implications, and the possible benefits and risks of participation. Written informed consent was obtained from all participants.

### Physical Activity and Sedentary Behavior Determination

Accelerometry was used to objectively measure physical activity and sedentary behavior. Accelerometers (Acti-Sleep V.3.4.2 accelerometer; Actigraph, Manufacturing Technology Inc., Shalimar, FL, United States) were worn on the dominant hand and data were recorded over 7 days (5 weekdays and 2 weekend days) except when water activities were performed. Energy expenditure and physical activity (light, moderate to vigorous, and vigorous activity) were calculated as previously described (Bressa et al., 2017). Sedentary behavior was characterized by the number of sedentary bouts (period of consecutive minutes where the accelerometer registers < 100 counts/min) and sedentary breaks (period of at least 1 min where the accelerometer registers ≥ 100 counts/min following a sedentary bout) and the total time spent in them (Byrom et al., 2016).

### Dietary Data

Diet habits and food intake were registered using self-reported food frequency questionnaire (FFQ). This questionnaire comprises 93 items, collects the annual food consumption and has been validated for the Spanish adult population (Vioque and Gonzalez, 1991; Vioque López, 2006; Supplementary Material). Data from the FFQ were analyzed using Dietsource software 3.0 (Novartis, Barcelona, Spain) to obtain the total energy ingested (in kcal) of proteins, fat, carbohydrates, fiber, and ethanol. Macronutrients data are expressed in grams (g) and as the percentage of the energy that they provide to the diet. Single foods were grouped in the following categories: fruits, vegetables, legumes, cereals, nuts, dairy products, white meat, red meat, processed meat, fish, eggs, pastries, and sugars.

### Body Composition Measurement

Body weight and height were measured with a scale and a stadiometer (Asimed T2, Barcelona, Spain). BMI was calculated by dividing the weight by the square of the height. Body composition was evaluated by dual-energy X ray absorptiometry (DEXA; GE Healthcare, Madison, WI, United States). Full DEXA scans were performed in supine decubitus position, and provided the following variables: total body fat mass, estimated visceral adipose tissue (VAT), total trunk fat mass, total body lean mass, and fat and lean mass for lower and upper limbs. The following indices were then calculated: adiposity index (AI) = total fat/height^2^; muscular mass index (MMI) = total muscle mass/height^2^ and appendicular muscular mass index (AppMMI) = muscle mass in arms + legs/height^2^, fat-free mass index (FFMI) = BMI – fat mass.

### Stool Collection, DNA Extraction, and Sequencing

Participants were provided with a Fe-Col^®^ (Alpha laboratories, Hampshire, United Kingdom) fecal sample collection kit to collect stool samples. Stool samples were collected once and were transported on ice to the laboratory where they were stored at −80°C until analysis. DNA was extracted using the commercial EZNA Stool DNA Kit (Omega Biotek, Madrid, Spain), following the manufacturer’s instructions, with a bead-beating homogenizer (Bullet BlenderStorm, Next Advance, New York, NY, United States) using glass beads for 3 min at speed 10 (following the manufacturer’s recommendations). The elution volume was 100 μL. The DNA concentration and purity of was measured using the Quant-iT PicoGreen dsDNA Assay Kit (Thermo Fisher Scientific, Waltham, MA, United States). Microbiota analyses were performed by amplifying the V3-V4 hypervariable regions of the bacterial 16S rRNA gene. The 459 base pair (bp) amplicon was visualized in a 0.8% agarose gel stained with ethidium bromide. The bands were cut and cleaned using the MinElute Gel extraction kit (Qiagen, Hilden, Germany). DNA amplicons were sequenced on a MiSeq Illumina platform (Illumina, San Diego, CA, United States). Raw data in fastq format have been deposited and are publicly available with the accession number PRJNA564612 the NCBI Biosample database^1^.

### Bioinformatics and Statistical Analysis

The 16S rRNA 2 × 300 pair-end reads were assembled and analyzed using the Quantitative Insights into Microbial Ecology (QIIME) program, version 1.9.1, using QIIME default parameters except for split library demultiplexing (phred quality threshold of 20 and better). The 16S rRNA pairend reads were assembled using the script multiple_join_paired_ends.py, which joins forward and reverse demultiplexed reads. High-quality sequences were grouped into operational taxonomic units (OTUs) with a sequence identity threshold of 97%; taxonomy was assigned by interrogating the high-quality sequences with the Greengenes database (13_8). The OTU table was rarefied to the minimum sample count (26979 sequences) for calculation of alpha-diversity metrics [Chao1, Phylogenetic Diversity (PD) tree, Shannon’s and Simpson’s indices] with the QIIME program. Beta-diversity was evaluated by calculating Bray-Curtis distance metrics. Principal component analyses (PCoA) of community structure (β- diversity) using the Bray-Curtis metric was generated by QIIME and analyzed by permutational multivariate analysis of variance (PERMANOVA) using the script comparecategories.py. The *p* values were corrected with the Bonferroni test.

### Microbial Core and Interactions Network

Network analysis is based on the estimation of the statistical relationship among bacteria abundances in order to infer or mutualism (positive correlation) or competition (negative correlation) per pair of bacteria. A network is composed by N nodes (N bacteria) connected by NxN links, where the strength refers to the weight of the correlation (*R* value) between pairwise bacteria abundances in a group (for a review see Faust and Raes, 2012; Layeghifard et al., 2017). The steps followed to build the network are presented below.

#### Data Preparation for Network Analysis

From the species classification level a core of bacteria was selected in order to consider only those taxa that were present in at least 75% of samples for each group (Faust et al., 2012). This core selection method aims to ensure a robust connectedness and avoid zero filling. In our dataset this core selection resulted in 55 OTUs for the ACT group, 52 for the SED group and 47 overlapping species in both groups (labels for core bacteria in Table 1). This core threshold retained an average of 95.41% of the identified species in each sample. The differential bacterial taxa between the two cores are shown in Supplementary Table S1. Raw OTU abundances were converted to relative values by normalization, namely by dividing each taxon abundance by the number of individuals in the community (Herren and McMahon, 2017).

**TABLE 1:** Overlapping bacterial taxa between active and sedentary individuals.

1	*Bifidobacterium longum*	25	Unclassified *Anaerostipes*
2	Unclassified *Coriobacteriaceae*	26	Unclassified *Blautia*
3	Unclassified *Bacteroides*	27	Unclassified *Coprococcus*
4	*Bacteroides caccae*	28	*Coprococcus eutactus*
5	*Bacteroides eggerthii*	29	Unclassified *Dorea*
6	*Bacteroides ovatus*	30	Unclassified *Lachnospira*
7	*Bacteroides plebeius*	31	Unclassified *Roseburia*
8	*Bacteroides uniformis*	32	*Roseburia faecis*
9	Unclassified *Parabacteroides*	33	*Ruminococcus gnavus*
10	*Parabacteroides distasonis*	34	*Ruminococcus torques*
11	Unclassified *Prevotella*	35	Unclassified *Ruminococcaceae*
12	Unclassified *Rikenellaceae*	36	Unclassified 2 *Ruminococcaceae*
13	Unclassified 2 *Rikenellaceae*	37	Unclassified *Anaerotruncus*
14	Unclassified *Barnesiellaceae*	38	*Faecalibacterium prausnitzii*
15	Unclassified *Butyricimonas*	39	Unclassified *Oscillospira*
16	Unclassified *Odoribacter*	40	Unclassified *Ruminococcus*
17	Unclassified *Streptococcus*	41	Unclassified *Mogibacteriaceae*
18	Unclassified *Clostridiales*	42	Unclassified *Erysipelotrichaceae*
19	Unclassified 2 *Clostridiales*	43	Unclassified *Holdemania*
20	Unclassified 2 *Christensenellaceae*	44	Unclassified *Sutterella*
21	Unclassified *Clostridiaceae*	45	Unclassified *Bilophila*
22	Unclassified *Clostridium*	46	Unclassified *Enterobacteriaceae*
23	Unclassified *Lachnospiraceae*	47	Unclassified *RF39*
24	Unclassified 2 *Lachnospiraceae*		

*The numbered bacteria correspond to the numbers shown in the interaction network figures.*

#### Pairwise Correlations

The data obtained for network estimation was an array of “number of bacteria” in the core (47, obtained from the previous step) per “number of individuals” (64 per ACT and 45 per SED), per group. In this work, we had two arrays: 47 × 64 for ACT, and 47 × 45 for SED. The network approach translates data into an interactions space with interest in identifying the relationship between individuals more than the individuals *per se*. In network terminology, the bacteria (*N* = 47) are the nodes of the network and the correlations between all of them are the symmetrical links composing the graph [(N^∗^N-1)/2; 1081 in this case]. The network structures were estimated using a Gaussian graphical model (GGM; Lauritzen, 1996). Due to the ordinal nature of the variables, a Spearman correlation matrix was used as input for the GGM (for a recent tutorial, see Epskamp et al., 2017). Spearman’s non-parametric rank coefficient estimates the linear correlation between all pairs of bacteria, given a symmetrical network, which can be either positive (co-occurrence) or negative (competition) depending on the direction of the correlation.

#### Network Building

The NxN matrix produced from the previous step provides the correlation between all pairs of bacteria. To convert the matrix into a network, it should be transformed in a semi-weighted matrix that is composed by positive (co-occurrence), negative (competition), or no correlation. Only the correlations that were statistically significant were used to construct the network. Significance was established at a *p*-value of 0.00001 because data were corrected by the Bonferroni test for multiple comparisons [number of connections in the network is 1081; 46^∗^(47/2)]. In order to individually study the role of each bacterial taxon in the network, we measured the degree and strength of nodes. Degree is the number of links connected to each node, and the strength is the sum of the weights of links connected to the node. These measurements can be used to identify the hubs of the network. The hub core constitutes the top 10% of the nodes. Topological measurements were estimated for both mutualism and competition networks. Transitivity coefficient is the frequency of loops of length three (clusters) in the network; it provides a measurement of the propagation (how continuous a network is). The Parcor R-package (Krämer et al., 2009) was used to implement the adaptive approach.

#### Network Statistics

To ensure that the topological microbial structure was not random, and can be compared between groups, we normalized the ACT and SED networks with those built from 1000 randomly reorganized networks that maintained the links distribution. In order to compare ACT and SED networks we followed a permutation test where 1000 networks are built from a subset of the original samples and then statistically compared pairwise with a *p* value threshold of 0.00001 (Bonferroni corrected). Additionally, an isomorphism comparison test was used to compare SED and ACT graphs. Two networks are isomorphic if there is a permutation of the nodes in SED that results in the same structure as in ACT.

#### Network Representation

Networks were visualized using the q-graph R-package (Epskamp et al., 2012) and the Fruchterman-Reingold algorithm (Fruchterman and Reingold, 1991). Plotting algorithms draw close those nodes with stronger and/or more connections; nodes with low centrality populate the periphery. Compared with regular plotting where bacteria are drawn following the label number, this algorithm allows a direct visualization of those bacteria with a more relevant role in the network.

#### Network Stability

This factor measures how network interpretation remains stable with less observations, in this case, a smaller number of individuals in both experimental groups. To study the stability of the networks, we followed the protocol of Costenbader and Valente (2003). We first calculated the bridge strength degree (BSD) (Payton, 2017) of the original network including the whole dataset, to be compared with the BSD of the networks constructed with several subsamples with percentages from 1 to 0.5 (half of the sample) for 25 iterations. The measure of the stability is represented by the coefficient of central stability (CS). We computed stability indices for both SED and ACT group networks in order to ensure the stability of our results with the dataset.

#### Cohesion

Cohesion metrics (Herren and McMahon, 2017), as individual measures of community complexity, analyze microbial community interconnectedness. Cohesion is the projection of the network into OTU abundances, and it is estimated by multiplying the semi-weighted matrix by the relative abundances. Subsequently, the cohesion metric is split into positive and negative values. Cohesion is thus an individual measure of the network complexity per bacteria, and it can be correlated with other parameters, including dietary data, physical activity, or body-composition measurements. The term complexity is defined in ecology to refer to the number and strength of connections in a food chain (May, 1972; Schoener, 1974). A high cohesion value can indicate that the community has many taxa that are responding simultaneously to external forces. Therefore, cohesion is a good candidate for correlation with physical activity and dietary habits. The bioinformatic pipeline is available on GitHub.

### Statistical Analysis

Statistical analyses were performed using SPSS software v.21 (SPSS, Chicago, IL, United States) and R. The normal distribution of variables was confirmed by using the Shapiro test. Student’s *t*-test was used to compare the variables that fulfilled the assumption of normality (body composition, physical activity and diet). Diversity indices were compared using the Mann–Whitney *U* test. Significance was established at *p* < 0.05. For multiple comparisons the Bonferroni multiple correction was applied. PERMANOVA was used to test differences in microbial community composition (β-diversity) across groups based on pairwise Bray-Curtis distance matrix. Differentially abundant bacterial taxa analysis was performed using analysis of composition of microbiomes (ANCOM) via the ANCOM 2.0 package in R 3.6.1, corrected for age and sex variables; significance was defined as *W* > 0.7. If not otherwise specified, the values shown are the mean and standard deviation.

## Results

### Characteristic and Lifestyle of the Participants

A cohort of 109 individuals was enrolled in the present study: 64 in the ACT group and 45 in the SED group. The mean age was 32.17 ± 7.40 years in the ACT group and 33.69 ± 7.96 in the SED group. There were no significant differences in BMI between the groups (ACT = 24.01 ± 3.28 kg/m^2^ and SED = 23.63 ± 2.91 kg/m^2^; *p* = 0.71). By contrast, there were marked differences between the SED and ACT individuals in all evaluated muscle and fat-related parameters (Table 2).

**TABLE 2:** Body composition of ACT and SED individuals.

	**ACT (*n* = 64)**	**SED (*n* = 45)**	***p*-value**
BMI (kg/m^2^)	24.013.28	23.632.97	0.549
BFP (%)	22.716.09	32.356.04	<0.001
AI (kg/m^2^)	5.301.76	7.531.96	<0.001
VAT (g)	274.39150.05	417.66196.62	<0.001
MMI (kg/m^2^)	16.881.93	14.662.14	<0.001
AppMMI (kg/m^2^)	7.651.14	6.361.18	<0.001
BFM (kg/m^2^)	15.655.71	21.335.543	<0.001

*BMI, body mass index; BFP, body fat percentage; VAT, estimated visceral fat; AI, adiposity index; MMI, muscular mass index; BFM, body fat mass; AppMMI, appendicular muscular mass index. Values are means ± standard deviation.*

As expected, there were clear differences between SED and ACT individuals regarding physical activity (Supplementary Table S2). An active lifestyle was reflected in almost all the determined parameters, except for light physical activity, in which there were no differences between groups. Energy expenditure was higher in the ACT compared to SED individuals. Furthermore, there were significant differences in the moderate and moderate-vigorous physical activity parameters. The SED group was characterized by less interruptions of sedentarism (sedentary breaks) and longer sedentary bouts.

Dietary habits differed between ACT and SED individuals (Supplementary Table S3). In terms of macronutrients, both diets were slightly imbalanced considering the appropriate percentage of energy provided by macronutrients as follows: 10–15% for proteins, 50–60% for carbohydrates and 30–35% for lipids (Martínez Álvarez et al., 2010). Further, lipid and carbohydrate consumption were higher in the SED group, whereas protein consumption was higher in the ACT group. When dietary habits were compared in terms of grams of macronutrients, there were no significant differences were detected except for fiber consumption. ACT individuals consumed higher amounts of fiber, a phenomenon explained by a greater consumption of fruits and nuts, whereas SED individuals consumed more processed meat and sugars.

### Diversity and Microbial Composition Analyses

**FIGURE 1:**
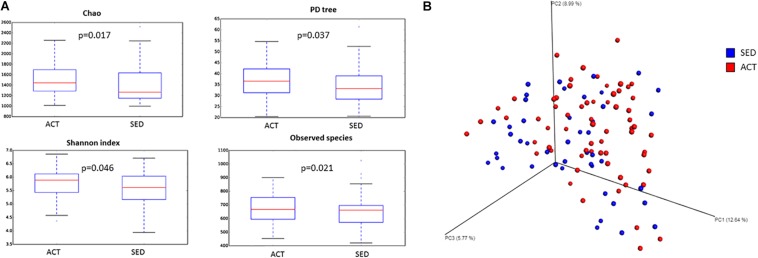
Differences in α-diversity (Chao, Shannon’s index, PD tree, and observed species) **(A)** and β-diversity (Bray-Curtis index) **(B)** between active (ACT) and sedentary (SED) groups. α-Diversity boxplots reflect median (horizontal center line), 25th and 75th percentile values (bottom and top bounds of boxes), and ranges (bottom and top of whiskers) for each category.

Rarefaction curves based on observed species, Shannon’s index, and phylogenetic distance measures were virtually saturated, findings that indicate sufficient sequencing depth (data not shown). The diversity of the two studied populations was estimated using qualitative (PD tree, Chao1) and quantitative (observed species, Equitability, and Shannon’s and Simpson’s indices) measures. Both qualitative and quantitative species-based measures were significant between groups with the exception of the Simpson’s index (*p* = 0.097) and Equitability (*p* = 0.086; Figure 1A). These data indicate that evenness was similar between the groups. The beta-diversity analysis showed both groups were significantly different (Figure 1B; PERMANOVA *p* = 0.020; pseudo *F* = 2.12). This finding suggests that microbiota samples from the same group were more similar to each other than to the other group. ANCOM revealed that SED individuals were characterized by a microbiota with a predominance of *Bacteroides* (ACT = 25.39%; SED = 32.23%) and *Parabacteroides* (ACT = 2.21%; SED = 2.88%) genera, while in the ACT microbiota exhibited a predominance of the *Coprococcus* (ACT = 3.49%;SED = 2.35%), *Blautia* (ACT = 3.89%; SED = 2.41%), and *Eubacterium* (ACT = 0.42%; SED = 0.29%) genera. Bacterial composition of the microbiota of both groups at phylum, family and genus level is shown in Supplementary Figure S1.

### Microbial Interactions Network in Active and Sedentary Lifestyle

**FIGURE 2:**
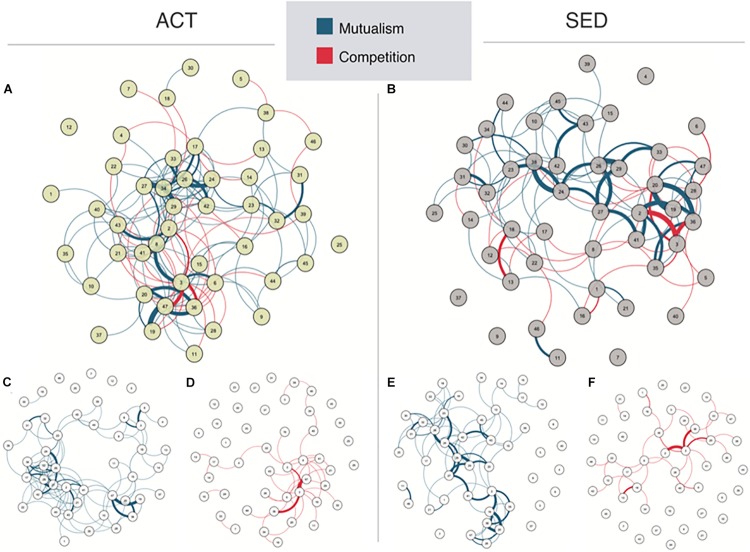
Network representations in **(A)** active (ACT) and **(B)** sedentary (SED) populations. This network is composed by nodes (circles representing the bacteria abundances named in Table 1) and edges (lines representing the statistical correlation between nodes). Blue edges represent positive relationships, while red edges represent negative relationships. The spatial position of the nodes was chosen by the Fruchterman-Reingold algorithm to draw close those nodes with stronger and/or more connections while placing with low centrality at the perifery. The network is split into mutualism and competitiveness networks [**(C,D)** for ACT, and **(E,F)** for SED, respectively].

Microbial networks were estimated for both ACT (*N* = 64) and SED (*N* = 45) groups, which included both positive and negative interactions, which could be distinguished based on the polarity of the correlation coefficients between the bacterial taxa core components. The graphical representation of the network showed that network reorganization depended on the lifestyle (Figures 2A,B). Both networks were topologically different according to the isomorphism comparison test. The ACT network had a modular behavior composed by one main bacterial cluster, whereas the SED group network exhibited a lower modularity and number of connections. To clearly differentiate the contribution of mutualism and competition networks to the microbial populations, we drew them separately (Figures 2C,F). Note that due to the nature of drawing algorithm, the network representation changes when mutualism and competition are separated, allowing a direct visualization of the more connected bacteria (drawn closer). When the competition ACT network (Figure 2C) was compared with the competition SED network (Figure 2E), there was a significant decrease in competitiveness, measured as a loss of the number of negative or competitive interactions (ACT = 1.97, SED = 1.54: average of interactions per bacterial taxon, *p* < 0.0001, Bonferroni corrected). The decrease in competitiveness in the SED group was based on the loss of negative interactions in the following microbial taxa: an unclassified species of *Bacteroides* genus, *Bacteroides ovatus* and *Bacteroides uniformis*, and an increase of competitiveness in an unclassified genus of the *Christensenellaceae* family.

Regarding mutualism (Figure 2D for ACT and 2F for SED), we also observed a non-significant tendency for reduction in the SED group (ACT = 4.66, SED = 3.40; average of interactions per bacterial taxa, not significant after Bonferroni correction). However, in this case, the reduction of positive connections was not localized in hubs or centers of modularity (the reduction of mutualism in the SED group was uniform when compared with the ACT group). The mutualism SED network was more spread than the ACT network, as measured by the increase in the transitivity coefficient (SED = 0.78, ACT = 0.44; *p* < 0.0001, Bonferroni corrected).

**FIGURE 3:**
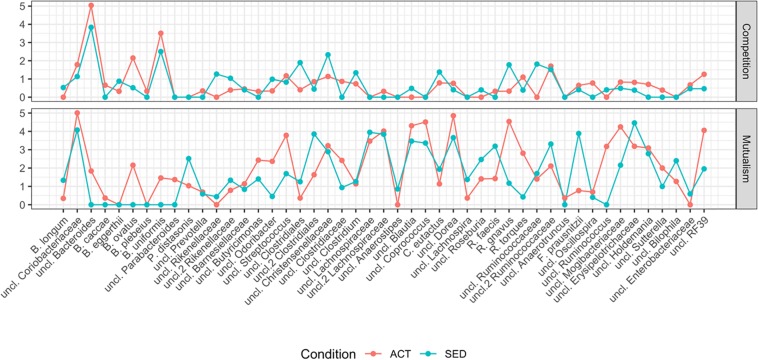
Topological characterization of ACT and SED networks measured by the number of connections (the higher the degree, the more connected the network is) per node (*x*- axis; full name description in Table 1) for competitiveness (top panel) and mutualism (bottom panel) networks.

Analysis of the degree of the network (number of connections per node; Figure 3), identifies the hub bacterial taxa (sets of nodes with the highest degree). Regarding mutualism (Figure 3, top panel), the hubs in ACT group were an unclassified species of the *Bacteroides*, *B. ovatus* and *B. uniformis*. In the SED network, the hubs were the same except without *B. ovatus*. The mutualism topological analysis revealed a modular behavior, where the highest degree was accumulated in a small subset of bacteria. Regarding competition (Figure 3, bottom panel), the degree was more homogeneous, and a subset of hubs could not be defined.

**FIGURE 4:**
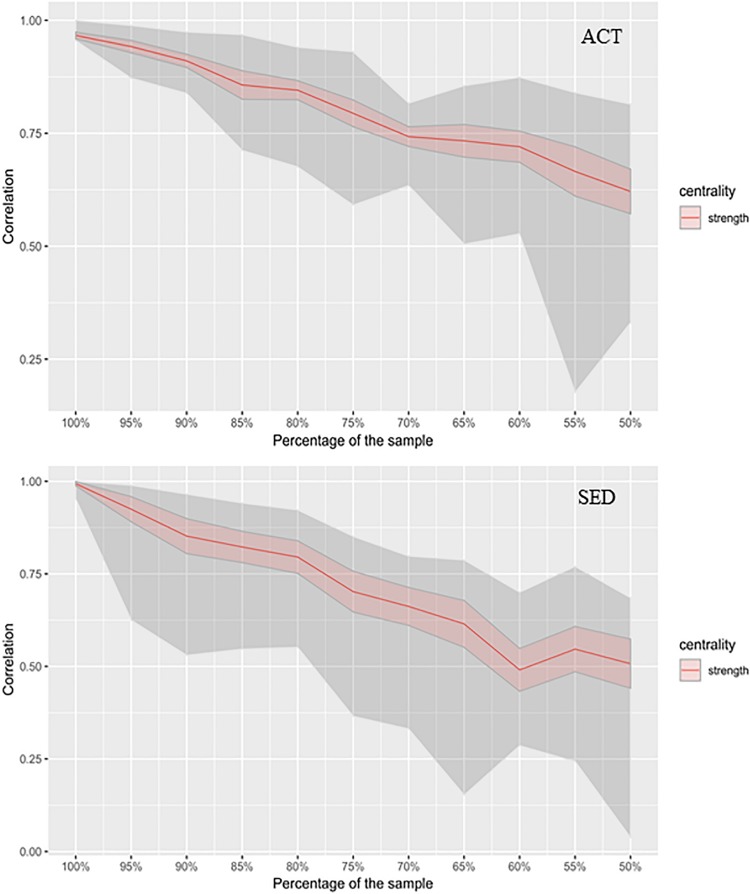
Stability as a measure of how network interpretation remains stable as the number of observations (sample size) decreases. The *x*-axis represents the percentage of participants considered for the subsamples (ACT and SED), from 100% (whole dataset) to 50% (half of the sample). *y*-axis represents the stability index measured by the strength of the network. The red area is the confident interval and the gray area is framed by the maximum and minimum values of the stability of the interactions per subsample (extreme values).

Network stability measures ensure that the datasets (number of subjects) of the groups are sufficient to provide a robust interpretation of the network. Thus, we computed stability indices for both SED and ACT networks in order to ensure the stability of our results with the dataset. Figure 4 shows the stability of centrality index strength, where the mean of the iterations (see section “Materials and Methods”), confidence intervals (red area), and extreme edges are represented. Figure 4 showed high stability along the bootstrapped samples (i.e., drops in stability were very small). The coefficient of CS indicated that strength centrality was moderately stable for both groups. One interpretation of the CS is that a subset composed by the 75% of the participants in SED group provides a network whose strength is 75% of the original network (composed by the complete dataset). However, in the ACT group a subset composed by the 75% of the subjects provides a network whose strength is 82% of the original. This finding implies that the ACT group was slightly more homogeneous compared to SED group. Both groups showed a low tendency of decrease in the strength with the reduction of the samples. Thus, we concluded that both were stable groups to infer the networks.

**FIGURE 5:**
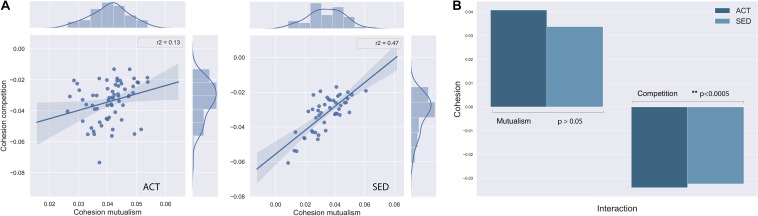
**(A)** Mutualism-competition complexity network relationship in ACT and SED groups, that shows network coupling in SED individuals. **(B)** Bar diagrams that compare mutualism and competition complexity averaged in both groups, where competitiveness was significantly different between SED and ACT individuals.

In order to obtain a measure of the complexity of the network and correlate it with diversity parameters, we computed the cohesion (see section “Materials and Methods” for definition) for both SED and ACT groups (Figures 5A,B). For the individual cohesion measure (Figure 5A), there was a decrease in competitiveness network complexity in the SED group (ACT = −0.034 ± 0.012, SED = −0.031 ± 0.01 (std); *p* [adjusted] = 0.00014; Figure 5B).Comparatively, the reduction in mutualism was not significant (ACT = 0.0410 ± 0.0068 SED = 0.032 ± 0.01; *p* [adjusted] = 0.355; Figure 5B). Interestingly, mutualism and competitive complexity were statistically correlated in the SED group (*R*^2^ = 0.47, *p* [adjusted] = 2.0 × 10^–7^), but not in the ACT group (*R*^2^ = 0.13, *p* [adjusted] = 0.02; Figures 5A,B). This coupling between mutualism and competitiveness in the SED group can be interpreted as a reduction in the network complexity, as discussed below. The mutualism-competitiveness complexity ratio (M-C ratio) was significantly higher for the ACT group (1.36 ± 0.62) compared to the SED group (1.19 ± 0.65).

### Network Complexity and Diversity

When correlations were established to study the relationship between the co-occurrence network – and microbial diversity, we found that the M-C ratio positively correlated in the SED group with PD tree (*R* = 0.67), Chao (*R* = 0.60), equitability (*R* = 0.53), observed species (*R* = 0.62), Shannon’s index (*R* = 0.66) and Simpson’s index (*R* = 0.45). In the ACT group, the M-C index correlated with Chao (*R* = 0.27) and observed species (*R* = 0.36). All correlations had *p* < 0.001 as a threshold.

## Discussion

Our results showed a decrease in the network complexity and a lower grade of competitiveness in sedentary compared to active individuals. These changes could, at least partly, represent the origin of the decreased diversity observed in a sedentary lifestyle. According with our results, previous studies described that a Western lifestyle may drive gut microbiota diversity depletion (De Filippo et al., 2010; Deehan and Walter, 2016; Menni et al., 2017). Active individuals who comply with the WHO recommendations for physical activity, and with a diet characterized with a higher fiber intake and low content of sugars and fats, presented a higher degree of competition (negative correlations) in three species of the *Bacteroides* genus: *B. ovatus*, *B. uniformis* and an unclassified species. *Bacteroides* is one of the most dominant genera of the core microbiota; it is considered to be a functional driver (Tito et al., 2018). This genus is characterized by high versatility for utilizing different glycan sources and its ability to adapt its metabolic machinery to the food source (Wexler, 2007). Moreover, *B. uniformis* produces short-chain fatty acids and the neurotransmitter GABA, whose production increases with complex glycans and could clearly affect the health of the host (Benítez-Páez et al., 2017). *Bacteroides* species could be a result of the top-down selection for functional redundancy because their genome contains a large number of genes involved in the acquisition, breakdown, or synthesis of carbohydrates (Ley et al., 2006a). Conversely, the *Christensenellaceae* family represents the bacterial taxon with a significantly greater number of negative interactions in the SED group. A higher abundance of this family has been associated with leanness, a high frequency of bowel movements (Oki et al., 2016), and a high capacity for running in ovariectomized rats (Liu et al., 2015). Nevertheless, more studies are needed to understand the implications of the negative interactions of the *Christensenellaceae* taxon in the SED group (that has the same BMI as the ACT group but a higher body fat composition) and their consequences.

Cooperation or competitiveness can usually be explained through comparison of phylogenetic versus functional similarities. Phylogenetically close taxa tend to positively associate with each other, whereas distantly related taxa with functional similarities tend to compete (Faust et al., 2012). Indeed, our mutualism analyses showed that *Bacteroides* species cooperate with each other as well as other species of phylogenetically close taxa, including *Parabacteroides* and *Odoribacter*. This cooperation is likely a result of functional redundancy that confers stability to the microbiota network, and slightly different preferences for substrates that result in metabolic cooperation (Ley et al., 2006a). Microbial communities with greater diversity have a greater number of positive and negative interactions (Faust et al., 2012).

Notably, our topological measure was based on the complexity of the network, also called cohesion (Herren and McMahon, 2017), and not simply the degree, strength, or number of connections. We chose a topological measure to extend the meaning of an intricate relationship. The decrease in the network cohesion occurs by affecting selective species, not in a uniform way but by decreasing the nested nature of the competitive network. Natural networks are proposed to not be random; but rather to display a nestedness or modular structure (Bascompte et al., 2003, 2006; Fortuna et al., 2010) that is related to the stability and dynamics of the ecosystem (Gracia-Lázaro et al., 2018). The loss of modularity or nestedness behavior generally entails a reduction in richness or topological efficiency (Toroczkai, 2017), as well as effective interspecific competition. These factors enhance the number of coexisting species (Bastolla et al., 2009).

Our study demonstrated that the network complexity of co-occurrence was higher than that for competition in both groups. However, both relationships were coupled in sedentary but not in active individuals. This coupling between networks can be interpreted as a loss of global complexity because it indicates dependence instead of coexistence or co-occurrence. The decrease in populations of competitive bacteria in the SED group would lead to a decrease and/or increase in other species, so the variances would be coupled. For the ACT group, having more competitive populations would be regulated by other species, so the variances would be coupled. This coupling also supposes that gut microbiome network reorganization is more predictable in the sedentary population. This phenomenon has important implications for treatment tests, because the variance of a subset of bacteria can predict the behavior of others. In the case, for example, of antibiotic intake, given the decrease of some bacterial species due to the effect of the drug, one could better predict what would happen to the other bacteria in the sedentary population. If there is more diversity, as in active population, there is a greater probability of finding more ways to recover the allostatic balance. Therefore, the variance of the species that are not affected by the drug will be less predictable. With reduced diversity, the ecosystem has less regulatory capacity because species can be controlled.

Our sedentary individual results are similar to another study, that examined co-occurrence in the microbiota of different sites of the human body, and detected a more balanced ratio of microbial co-occurrence versus co-exclusion interactions (Faust et al., 2012). Additionally, a positive correlation between the number of co-occurrence partners and environmental diversity has been previously observed (Freilich et al., 2010). The results from our study suggests that sedentary lifestyle and diet are associated with significantly reduced diversity and less dense microbial network structure with lower density connections. Furthermore, the level of competitive network interactions was significantly higher in fecal samples from active individuals. We postulate that a high M-C ratio is associated with a rich diversity where both networks co-exist. To study the relevance of the relationship between mutualism and competitiveness complexity, we correlated the M-C ratio with diversity parameters. There was a positive correlation with diversity parameters, including PD tree, Chao index, equitability, observed species, and Shannon’s and Simpson’s indices in both the ACT and SED groups. The positive nature of these correlations highlights the importance of a non-balanced ratio (>1), where mutualism is more abundant than competition, but under an equilibrated coexistence. The M-C ratio was less correlated with diversity measures in active compared to sedentary individuals. This finding suggests that other factors drive microbial diversity in active individuals. These additional factors (not considered in this study) could include food timing or fasting which could impact on substrate availability and therefore on the spatial conformation of the population history of antibiotic use, growth rate, and motility of the bacteria populations or bacteria communication (Quorum sensing mechanisms) (Hibbing et al., 2009; Levy and Borenstein, 2013). A better understanding of the complex interplay between mutualism and competitiveness may help guide clinical studies, such as the establishment of the optimal value of the M-C ratio that maximizes diversity or guarantees stability and resilience against threats or antibiotics.

## Data Availability Statement

The datasets generated for this study can be found in the NCBI Biosample database: https://www.ncbi.nlm.nih.gov/bioproject/564612.

## Ethics Statement

All procedures performed in studies involving human participants were in accordance with the ethical standards of the Institutional National Research Committee (Clinical Research Ethics Committee of the Ramón y Cajal Hospital in Madrid, reference 384-14) and with the 1964 Helsinki declaration and its later amendments. The participants provided their written informed consent to participate in the study.

## Author Contributions

NC, GD, CA-A, and ML conceived and designed the experiments. CB, MB, ML, NC, MP, and RG performed the experiments and analyzed the data. NC and ML wrote the manuscript.

## Conflict of Interest

The authors declare that the research was conducted in the absence of any commercial or financial relationships that could be construed as a potential conflict of interest.

## Footnotes

1 https://www.ncbi.nlm.nih.gov/bioproject/564612
