# Supplementary figures and images for: A Critical Mutualism – Competition Interplay Underlies the Loss of Microbial Diversity in Sedentary Lifestyle
Source: Front Microbiol. 2020 Jan 22;10:3142. doi: 10.3389/fmicb.2019.03142 (PMC6987436; doi:10.3389/fmicb.2019.03142)

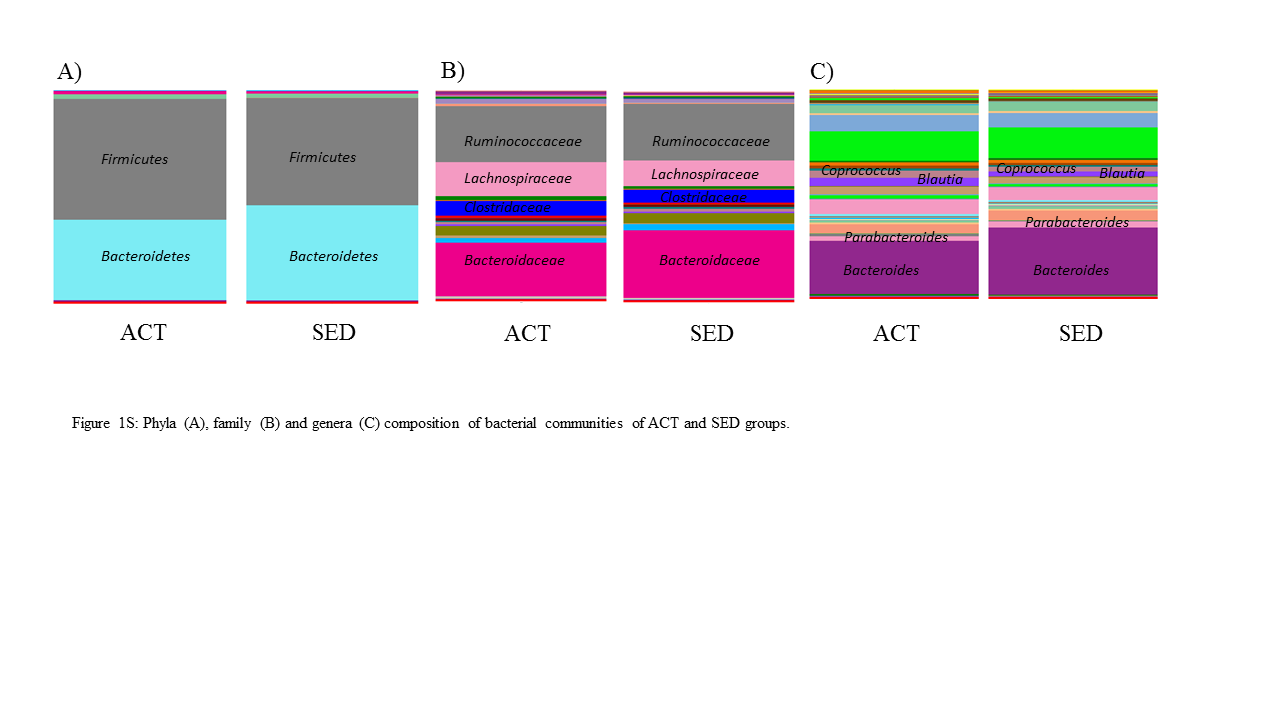

Supplement: Supplementary file 1 [file Image_1.TIF]
