# Supplementary material for: A Critical Mutualism – Competition Interplay Underlies the Loss of Microbial Diversity in Sedentary Lifestyle
Source: Front Microbiol. 2020 Jan 22;10:3142. doi: 10.3389/fmicb.2019.03142 (PMC6987436; doi:10.3389/fmicb.2019.03142)
Supplement: Supplementary file 2 [file Data_Sheet_1.PDF]

Table S1 Differential bacterial taxa between the two bacterial cores (ACT and SED)

**ACT**	**SED**
unclassified *S24-7*	unclassified *Bacteroides*
unclassified *CF231*	unclassified *RF32*
unclassified *Turicibacter*	unclassified *Desulfovibrionaceae*
unclassified *Coprococcus*	*Akkermansia muciniphila*
unclassified *Ruminococcus*	
Veillonella dispar	
Haemophilus parainfluenzae	

ACT: active individuals; SED: sedentary individuals.

Table S2 Physical activity and sedentary behavior of the study individuals

	**ACT (n=64)**	**SED (n=45)**	**p value**
Energy expenditure (kcal)	1909.01 ± 657.70	1402.30 ± 680.86	0.002
LPA (min)	769.29 ± 384.94	826.90 ± 244.80	0.093
MPA (min)	371.07 ± 114.71	298.35 ± 115.59	0.007
MVPA (min)	286.08 ± 110.01	214.59 ± 90.97	0.003
Sedentary breaks/day	16.14 ± 3.55	14.68 ± 2.62	0.013
Total time sedentary breaks (min/day)	665.74 ± 154.44	573.56 ± 172.53	0.015
Sedentary bouts/day	14.84 ± 2.62	16.79 ± 3.51	0.008
Total time sedentary bouts (min/day)	298.66 ± 64.29	340.62 ± 51.13	0.002

ACT: active individuals; SED: sedentary individuals; LPA: light physical activity; MPA: moderate physical activity; MVPA: moderate-vigorous physical activity. Values are means ± standard deviation.

Table S3 Dietary habits in study participants

DIET	**ACT (n=64)**	**SED (n=45)**	**p value**
Total energy (kcal)	2197.87 ± 751.44	2163.75 ± 673.97	0.814
Energy from carbohydrates (%)	44.07 ± 4.93	47.38 ± 6.93	0.009
Energy from protein (%)	17.97 ± 2.86	16.43 ± 2.64	0.006
Energy from fat (%)	34.65 ± 6.56	39.48 ± 5.23	<0.001
Carbohydrates (g)	258.04 ± 94.31	232.23 ±76.71	0.146
Fiber (g)	28.11 ± 13.21	19.33 ±7.05	<0.001
Proteins (g)	98.29 ± 44.20	85.94 ±28.33	0.114
Lipids (g)	82.75 ± 31.00	93.03 ±33.39	0.146
-saturated (g)	23.04 ± 9.86	24.95 ±9.21	0.330
-monounsaturated (g)	36.43 ± 14.02	38.89 ±14.06	0.426
-polyunsaturated (g)	9.00 ± 3.77	9.11 ±3.56	0.992
Ethanol (g)	3.65 ± 3.41	3.66 ±3.82	0.988
Fruits (s/d)	2.94 ± 2.11	1.73 ±1.18	0.001
Vegetables (s/d)	3.11 ± 2.06	2.69 ±1.24	0.240
Legumes (s/d)	0.22 ± 0.20	0.22 ±0.16	0.802
Cereals (s/d)	2.38 ± 1.44	1.93 ±1.09	0.101
Nuts (s/w)	2.69 ± 2.68	1.69 ±1.89	< 0.001
Dairy products (s/d)	2.41 ± 1.48	2.20 ±1.37	0.474
White meat (s/w)	3.04 ± 2.23	2.59 ±1.32	0. 253
Red meat (s/w)	2.76 ± 2.63	2.96 ±1.25	0.658
Processed meat (s/w)	1.22 ± 0.44	2.28 ±0.89	<0.001

Fish (s/w)	6.19 ± 4.80	4.18 ±1.98	0.022
Eggs (units/w)	3.03 ± 1.29	2.80 ±1.30	0.391
Pastries (s/w)	8.35 ± 11.27	8.35 ±9.18	0.898
Sugars (s/d)	0.36 ± 0.48	0.91 ±0.90	0.001

ACT: active individuals; SED: sedentary individuals; s/d: servings per day: s/w: servings per week
